# Electron-band theory inspired design of magnesium–precious metal bulk metallic glasses with high thermal stability and extended ductility

**DOI:** 10.1038/s41598-017-03643-7

**Published:** 2017-06-13

**Authors:** Kevin J. Laws, Karl F. Shamlaye, Davide Granata, Leah S. Koloadin, Jörg F. Löffler

**Affiliations:** 1 Laboratory of Metal Physics and Technology, Department of Materials, ETH Zurich, 8093 Zurich, Switzerland; 2 School of Materials Science and Engineering UNSW Australia, NSW, 2052 Australia

## Abstract

Magnesium-based bulk metallic glasses (BMGs) exhibit high specific strengths and excellent glass-forming ability compared to other metallic systems, making them suitable candidates for next-generation materials. However, current Mg-based BMGs tend to exhibit low thermal stability and are prone to structural relaxation and brittle failure. This study presents a range of new magnesium–precious metal-based BMGs from the ternary Mg–Ag–Ca, Mg–Ag–Yb, Mg–Pd–Ca and Mg–Pd–Yb alloy systems with Mg content greater than 67 at.%. These alloys were designed for high ductility by utilising atomic bond-band theory and a topological efficient atomic packing model. BMGs from the Mg–Pd–Ca alloy system exhibit high glass-forming ability with critical casting sizes of up to 3 mm in diameter, the highest glass transition temperatures (>200 °C) of any reported Mg-based BMG to date, and sustained compressive ductility. Alloys from the Mg–Pd–Yb family exhibit critical casting sizes of up to 4 mm in diameter, and the highest compressive plastic (1.59%) and total (3.78%) strain to failure of any so far reported Mg-based glass. The methods and theoretical approaches presented here demonstrate a significant step forward in the ongoing development of this extraordinary class of materials.

## Introduction

Magnesium and its alloys exhibit the lowest density of all engineering metals and continue to play an increasingly significant role in aerospace, automotive, consumer electronics and biomedical applications as a result of their high specific strength, stiffness and biocompatibility. Compared to crystalline alloys, amorphous alloys (metallic glasses) possess superior strength, elastic limits and corrosion resistance as a result of their homogeneous glassy structure^1, 2^. They also exhibit a broad range of fracture toughness properties, ranging from extremely brittle to some of the toughest materials known to date^3–5^.

Since their discovery in 1977^6^ numerous Mg-based amorphous alloys and subsequent multi-component bulk metallic glasses (BMGs) have been developed. The majority of these are based on Mg–TM–RE or Mg–TM–Ca ternary systems (where TM = transition metals Cu, Ni, Zn, Ag and RE = Y, Gd, La, Nd, Ce, etc.)^7–14^. Some show exceptional glass-forming ability with critical casting sizes up to 27 mm^15^, placing them amongst the highest glass-forming alloy families. However, Mg-based BMGs have yet to realise their full potential in long-term load bearing or functional applications due to their intrinsically brittle nature and relatively short structural relaxation times^16, 17^. To date, modest Mg-based ‘bulk’ glass ductility has only been observed in Mg-rich Mg–Ni–[Gd,Nd]^9, 10^, and Mg–Ni–Ca^14^ alloy systems. It has been postulated that the degree of electronic charge transfer between constituent elements (i.e. the degree of covalency in atomic bonds) needs to be minimised to improve ductility^16, 18, 19^, and the glass transition temperature raised in order to slow ambient-temperature structural relaxation﻿﻿ which also leads to embrittlement﻿﻿^16^.

In comparison, plasticity in pure or low-solute polycrystalline alloys results from atoms easily gliding past one another via dislocation-type mechanisms^20^. From an electronic bonding perspective this atomic translation is relatively easy, as the core charges of the metal ions are uniform and generally only involve outer shell electrons, with little charge transfer or bond directionality (covalency). Hence, atoms need to only move from one crystal lattice site to the next by overcoming a low-energy barrier before reaching an identical neighbouring site^20–22^. In a multi-component metallic glass, plasticity occurs via local shear transformations^23, 24^ (similarly, atoms must glide past one another to neighbouring structural sites). However, depending on the atoms present, complex intra-electron orbital bonding between dissimilar constituents may occur. This often results in considerably stronger and/or more directional (covalent) bonding between atoms through electron band hybridisation^18, 19, 25^. It is well-known that as the number of electrons involved in a hybridised bond increases, the energy/strength of this bond increases (i.e. in the order s-, p-, d- and f-electron bands) and in the case of p-, d- and f-bands, bonding is highly directional. Hence when atoms of different ion-core charges in a multicomponent metallic glass try to glide past one another during shear, the energy required is much higher and often directionally dependent. This results in a considerably altered mechanical response in glasses during deformation when compared to pure (crystalline) metals and is expected to be more closely akin to that of high-strength (covalent) intermetallics^21, 22^.

In this study we have applied a novel approach to developing Mg-based BMGs with high glass-forming ability and ductility. We implemented a predictive topological model^26, 27^ to locate the maximum glass-forming region of Mg-rich metallic systems of interest. This was combined with the anticipated electronic bonding states/interactions between constituents that minimise bond directionality, allowing atoms to be sheared more uniformly during deformation^21, 22^ in order to maximise glass ductility.

## Alloy Design Concept

It has been shown that a higher solvent content minimises covalent bonding and improves ductility in Mg-based glasses^16, 19^, hence higher Mg-contents are preferred. Despite their relatively high density and costs, precious metals exhibit exceptional properties with respect to ductility in BMGs. Some of the toughest materials known are Pd- and Pt-rich metallic glasses^4, 5^. When alloyed with another metal, elements that have a completely- or near-filled d-band require few if any electrons to fill the band and hence it tends to remain closer to the core of the atom (below the Fermi level, see ref. 28 for band locations of individual elements), contributing less to bonding^22^. Bonding in these alloys is primarily due to s-band interactions, which have no directionality and lower bond energy, thus during deformation atoms can glide past one another with relative ease leading to ‘more metallic’ or ductile behaviour. This bonding nature is also responsible for the bulk and shear moduli of these elements (i.e. their high Poisson’s ratio: Ag = 0.37, Au = 0.4, Pd = 0.39, Pt = 0.38), which are critical to shear-band branching and crack-shielding mechanisms in metallic glasses^4, 5, 23, 29^.

Recently, Laws *et al*. reported bulk glass-formation in the Mg–Ag–Ca ternary system in the Mg-rich region^13^, highlighting this as a potential precious-metal-containing composition space for ductile Mg-based glasses. Further, it has been found that the MgAg intermetallic compound exhibits considerable ductility, shown to be due to the full d-electron shell of Ag remaining below the Fermi level when bound to Mg^22^. The Mg–Pd binary alloy system^30^ exhibits similar attributes to Mg–Ag, with an eutectic reaction at ~17 at.% Pd (the same concentration as the Mg-rich Mg–Ag eutectic reaction^31^). Glass formation has also been reported in the binary Mg–Pd system for a composition range of 10–35 at.% Pd^32^. Based on this, Ag and Pd were selected as precious-metal additions with minimal d-band electron interactions for ductile glass development.

Ca is a large, chemically active alkaline metal (atomic radius ~197 pm) with an electronic configuration 1s^2^ 2s^2^ 2p^6^ 3s^2^ 3p^6^ 4s^2^ (no d-electron band in its ground state), effective in improving glass-forming ability in Mg-based glasses^12–14^. Yb is a unique rare earth/lanthanide metal that can exhibit a full f-electron shell and the stable oxidation state of +2 ([Xe] f^14^6s^2^), making it more closely akin to Ca. The 4d-electron band of Yb resides beneath the 5s, 5p and 4f-bands, respectively, and is not expected to interact in these alloys (the 5d band is empty). It is also known that 4f electrons have a high probability of being close to the nucleus of an atom or ion, resulting in little overlap between the orbitals of lanthanides and binding ions^33^. Similar to Ca, Yb has a large atomic radius of ~194 pm when adopting the full f-electron shell state and Yb has been shown to behave similarly to Ca with respect to glass-forming ability in other Mg-based systems^34^. Based on this, Ca and Yb were selected as potential alloying elements.

The efficient packing of atoms in an amorphous structure is known to hinder atomic diffusion upon cooling, retarding crystallisation kinetics, leading to greater glass-forming ability^13, 26, 27^. A predictive topological model (method described in detail in ref. 27) that determines composition space where efficient packing around each atom is achieved was utilised for alloy development. This model uses a ‘hard sphere’ approximation where, based on the alloy composition and the radii of the elements present, every atom in a given atom’s first coordination shell touches that central atom and its nearest neighbours, hence achieving global packing efficiency. Based on these concepts, a range of Mg-rich ternary alloys from the Mg–Ag–Ca, Mg–Ag–Yb, Mg–Pd–Ca and Mg–Pd–Yb systems were prepared and analysed.

## Results and Analysis

**Figure 1:**
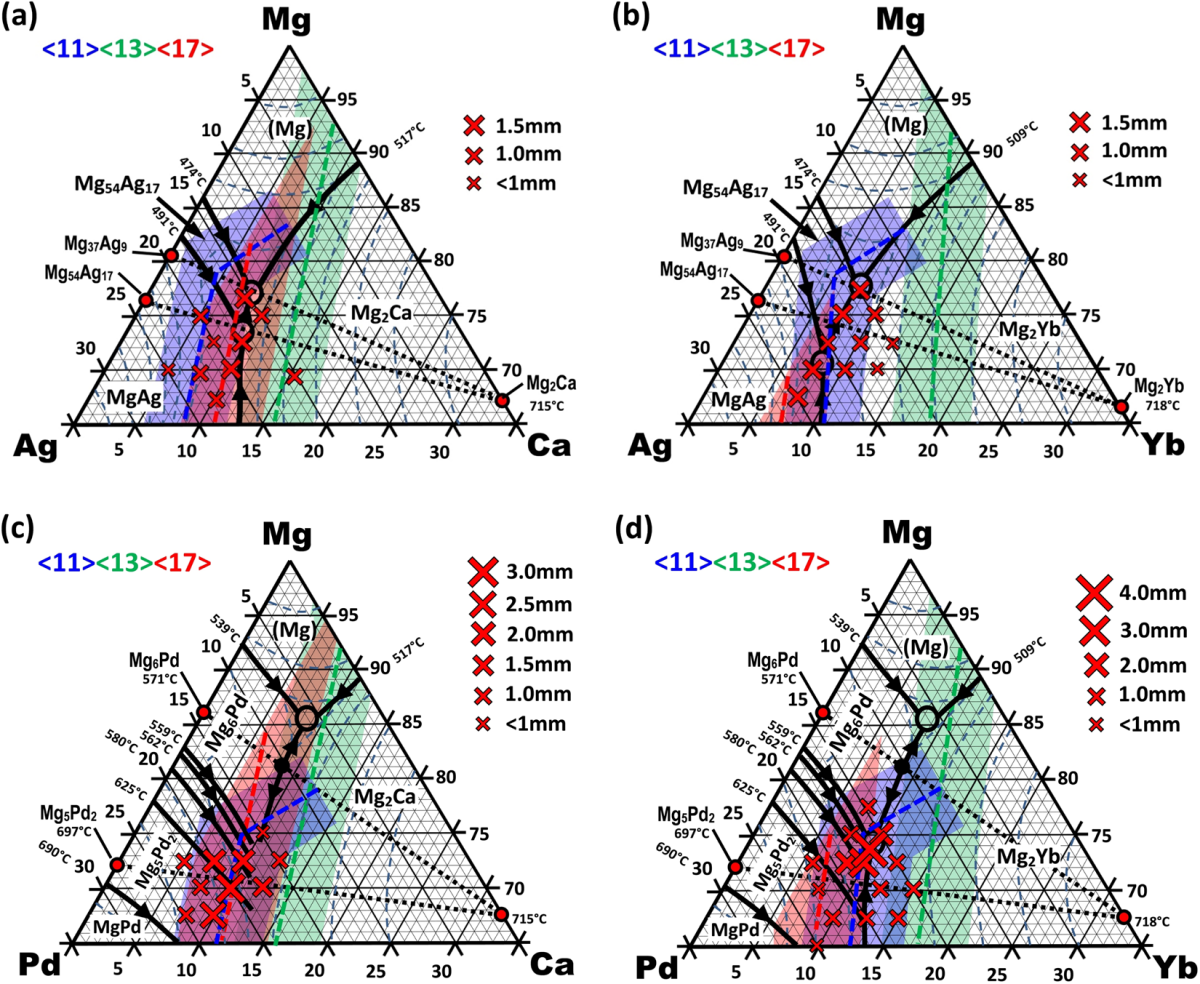
Ternary liquidus iso-surfaces of the Mg-rich regions in the (**a**) Mg–Ag–Ca, (**b**) Mg–Ag–Yb, (**c**) Mg–Pd–Ca, and (**d**) Mg–Pd–Yb alloy systems, determined using DSC data and binary phase diagram information^30, 33, 35, 36^. Also shown are the glass-forming alloy compositions tested, their relative glass-forming abilities (critical casting diameters) and the efficiently-packed cluster regions determined, where blue indicates Ag- or Pd-centred clusters, green Mg-centred clusters and red Ca- or Yb-centred clusters.

Figure 1(a–d) shows the Mg-rich composition space tested within this study for the Mg–Ag–Ca, Mg–Ag–Yb, Mg–Pd–Ca and Mg–Pd–Yb ternary alloy systems. Red crosses indicate the compositions of the alloys produced, and their size indicates their relative glass-forming ability. From binary system information^30, 31, 35, 36^ and DSC data generated from this work, a ternary liquidus iso-surface was produced for each system. Liquidus lines are indicated in black and ternary eutectics in hollow black circles. Also indicated are the precise efficient packing zones/compositions for each atomic centre. The atomic radii used for these calculations were Mg = 160 pm, Ag = 144 pm, Pd = 139 pm, Ca = 197 pm, and Yb = 194 pm^37, 38^.

Using the predictive topological model, it was found that Ag- and Pd-centred clusters can be efficiently packed in the Mg-rich region when they have a coordination number of <11> (indicated by the dashed blue lines). There is also a possibility of structural medium-range order (MRO) in this system. According to the efficient packing model, specific structural sites exist between independent clusters of atoms when these clusters are organised in a face-centred cubic (*fcc*) packing array^27, 39, 40^. These sites, defined as beta- (*β*) and gamma- (*γ*) sites, can accommodate smaller-sized atoms. When the *β*-site is filled by the same atomic species as the atom in the centre of a cluster and this is repeated multiple times in such an array, a degree of structural medium-range order develops that is known to further stabilise the glassy structure (see refs 27, 39 and 40. for in-depth details of this method). Mathematically, the *β*-site of an Ag- or Pd-centred cluster can be completely filled by Ag or Pd, respectively, at the ideal concentration of 16.67 at.% Ag or Pd, generating this favoured MRO sequence. Coincidentally, this composition corresponds precisely to binary eutectic reactions in both Mg–Ag and Mg–Pd systems^30, 31^, (eutectics and liquidus lines are a known compositional locality of liquid stability). This progressive *β*-site filling around Ag- and Pd-centred clusters is responsible for the ‘kink’ in the blue packing-efficiency regions in Fig. 1. In an almost identical composition space, Ca- and Yb-centred clusters that correspond to the nominal alloy composition are also efficiently packed when they have a coordination number of <17> (indicated by the red dashed lines); based on their size and constitution, their beta site can be filled with a Mg atom, further enhancing packing efficiency. Mg-centred clusters are efficiently packed with a coordination number of <13> (indicated in green) and can also achieve MRO with Mg in their *β*-site. This <11> <13> <17> coordination number combination is one of those preferred for glass formation based on ref. 26. The width of the associated efficient-packing coloured shaded areas corresponds to a deviation of ±1% in radius ratio, which allows for discrepancies in reported atomic radii in metallic solutions.

Table 1 gives the critical casting thicknesses (*Z*_*C*_) and diameters (*D*_*C*_) of the Mg–Ag–Ca alloys tested in this work and in ref. 13. Also given are their associated thermophysical properties: the glass transition temperature (*T*_*g*_); crystallisation onset temperature (*T*_*X*_), onset melting temperature (*T*_*m*_) and liquidus temperature (*T*_*l*_), and Vickers hardness. There is a clear correlation between BMG formation and the calculated efficiently-packed cluster regions, with the largest glass-formers found almost directly on the Ca-centred <17> coordination line. These larger BMG alloys and the <17> line also correspond closely to the ternary eutectic and peritectic reactions and the major liquidus trough of this system. This phenomenon is also reflected in the system’s glass-forming sensitivity to Ca-content and the complete beta-site filling of the Ag-centred clusters (at 16.67 at.% Ag). Notably, no glasses were found to exceed a critical casting size of 2 mm, although it was shown in earlier work that the quaternary addition of Cu increased the glass-forming ability to a critical casting diameter of 4 mm^13^.

**Table 1:** BMG-forming compositions in the Mg-rich region of the Mg–Ag–Ca ternary system, and their associated critical casting sizes, thermophysical properties, and Vickers hardness.

Composition	*Z*_*C*_ (mm)	*D*_*C*_ (mm)	^*^ *T*_*g*_ (°C)	*T*_*X*_ (°C)	*ΔT*_*X*_ (°C)	*T*_*m*_ (°C)	*T*_*l*_ (°C)	*HV*
Mg_76.9_Ag_15.4_Ca_7.7_^[13]^	1.1	1.7	125	141	16	400	432	240
Mg_75_Ag_15_Ca_10_	0.7	1.0	132	146	14	404	432	210
Mg_75_Ag_20_Ca_5_	0.8	1.0	142	155	13	416	442	219
Mg_72.5_Ag_17.5_Ca_10_	1.1	1.5	135	152	17	416	435	261
Mg_72.5_Ag_20_Ca_7.5_	0.5	—	155	167	12	415	446	259
Mg_69.2_Ag_15.4_Ca_15.4_^[13]^	0.3	—	—	—	—	408	519	—
Mg_70_Ag_20_Ca_10_	1.0	1.5	143	156	13	416	439	227
Mg_69.2_Ag_23.1_Ca_7.7_^[13]^	1.0	1.5	129	155	16	412	440	—
Mg_70_Ag_25_Ca_5_	0.5	—	—	—	—	—	—	—
Mg_67.5_Ag_22.5_Ca_10_	0.8	1.0	148	162	14	415	437	224
Mg_61.5_Ag_23.1_Ca_15.4_^[13]^	0.7	1.0	138	168	30	426	595	—

^*^ *T*_*g*_ values quoted here are the onset temperatures of the glass transition.

**Figure 2:**
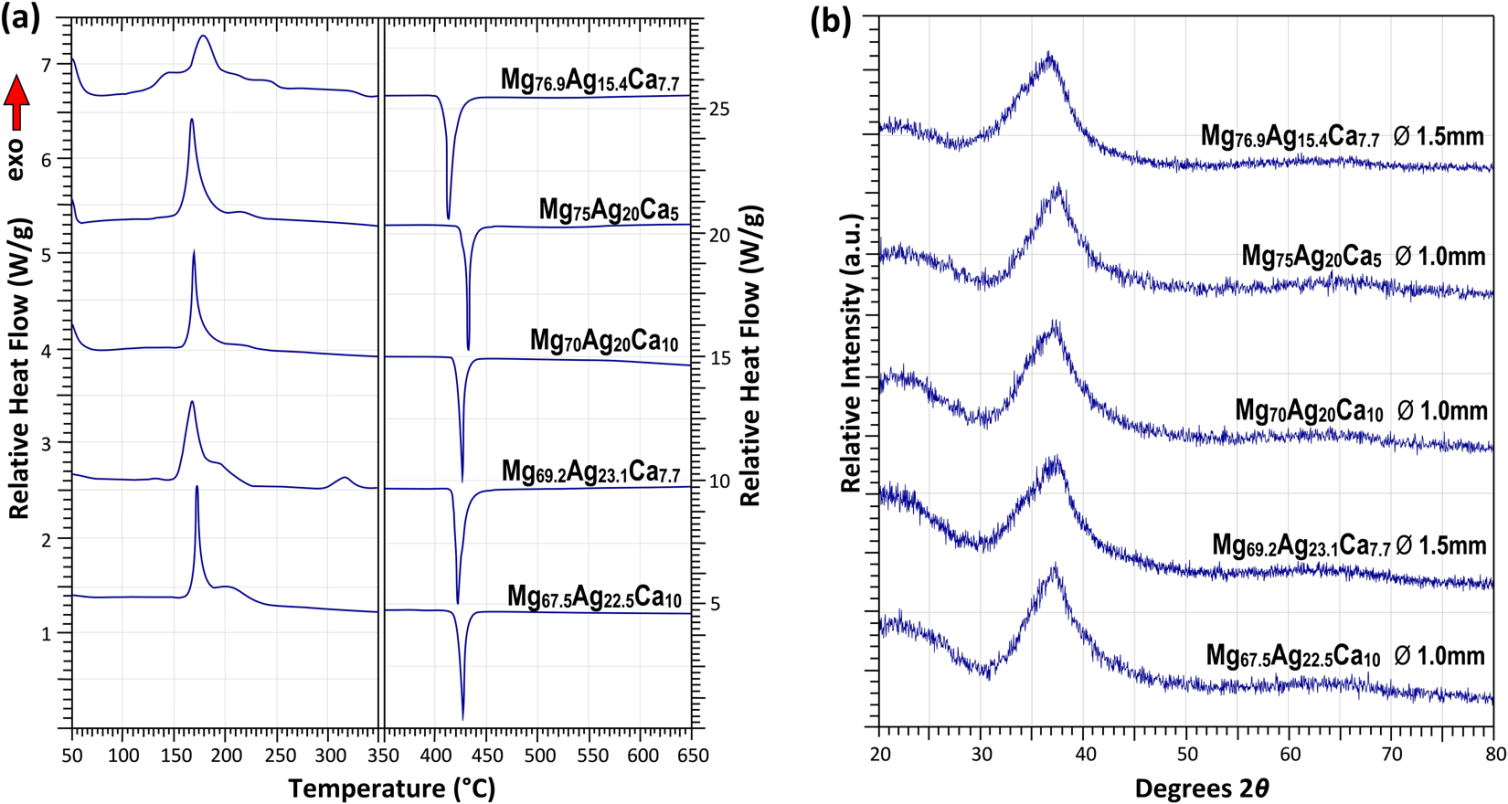
Selected (**a**) DSC and (**b**) XRD traces of Mg–Ag–Ca BMGs.

Figure 2(a) shows selected DSC data of Mg–Ag–Ca BMGs. A clear glass transition and proceeding crystallisation reactions can be seen in each case. It is also notable that compositions with a Mg-content greater than 70 at.% and Ca-content less than 10 at.% begin to show a more pronounced exothermic structural relaxation upon approaching the glass transition than the other alloys. It was also observed that these alloys displayed more ductile-like behaviour during handling and sample preparation than those of lower Mg- and higher Ca-content. The liquidus data suggest that all of these alloys are in close proximity to a single peritectic and eutectic reaction, which is depicted in Fig. 1(a). It is also clear that the crystallisation sequence is altered significantly as compositions approach the ternary eutectic reaction, with *α*-Mg becoming more clearly the first crystallisation reaction product for the Mg_76.9_Ag_15.4_Ca_7.7_ alloy. Figure 2(b) shows XRD traces for these glasses, exhibiting the characteristic diffuse halo of an amorphous structure.

Table 2 lists the *Z*_*C*_ and *D*_*C*_ values, and the thermophysical and hardness properties of the Mg–Ag–Yb alloys produced and tested. The efficiently-packed composition space in this system is slightly different from that of the Mg–Ag–Ca system, shifting to lower Yb (large atom) concentrations, which results from the slightly smaller radius of Yb opposed to Ca. The glass-forming maxima and liquidus line (both around 7.5 at.% Yb) has also shifted accordingly. It is also worth noting that the overlay of the <11> <13> <17> efficient packing lines is slightly more spread compared to that of the Mg–Ag–Ca system. This suggests a poorer packing efficiency of the global structure, which may account for the lower GFA yet broader glass-forming composition range observed.

**Table 2:** BMG-forming compositions in the Mg-rich region of the Mg–Ag–Yb ternary system, and their associated critical casting sizes, thermophysical properties, and Vickers hardness.

Composition	*Z*_*C*_ (mm)	*D*_*C*_ (mm)	^*^ *T*_*g*_ (°C)	*T*_*x*_ (°C)	*ΔT*_*X*_ (°C)	*T*_*m*_ (°C)	*T*_*l*_ (°C)	*HV*
Mg_76.7_Ag_15.4_Yb_7.7_	1.1	1.7	107	146	39	403	426	240
Mg_75_Ag_15_Yb_10_	0.8	1.0	122	146	24	403	431	246
Mg_75_Ag_17.5_Yb_7.5_	1.0	1.5	123	155	32	403	433	246
Mg_72.5_Ag_15_Yb_12.5_	0.5	—	147	176	29	402	478	248
Mg_72.5_Ag_17.5_Yb_10_	0.7	1.0	132	165	33	406	437	205
Mg_72.5_Ag_20_Yb_7.5_	1.0	1.0	109	156	45	410	434	253
Mg_70_Ag_17.5_Yb_12.5_	0.6	—	—	174	—	409	460	—
Mg_70_Ag_20_Yb_10_	0.9	1.0	142	170	28	409	434	240
Mg_70_Ag_22.5_Yb_7.5_	1.1	1.5	129	180	51	409	436	257
Mg_70_Ag_25_Yb_5_	<0.5	—	—	—	—	400	513	—
Mg_67.5_Ag_22.5_Yb_10_	0.6	—	139	172	33	411	438	—
Mg_67.5_Ag_25_Yb_7.5_	1.0	1.5	125	182	57	407	433	269

^*^ *T*_*g*_ values quoted here are the onset temperatures of the glass transition.

**Figure 3:**
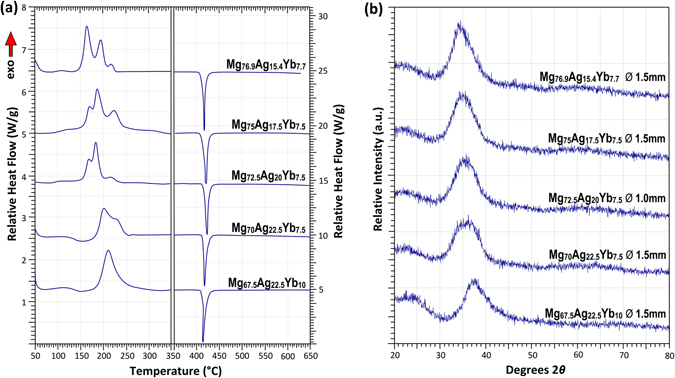
Selected (**a**) DSC and (**b**) XRD traces of Mg–Ag–Yb BMGs.

Figure 3(a) shows selected DSC data of Mg–Ag–Yb BMGs. Strong glass transition signals are evident for alloys with Mg-contents of 70 at.% and below. The supercooled liquid (SCL) interval (Δ*T*_*X*_) of the Mg_67.5_Ag_25_Yb_7.5_ alloy is around 57 °C, indicating a high resistance to devitrification, comparable to that of the very stable Mg–Cu–Y amorphous alloys (Δ*T*_*X*_~50–70 °C)^7^. This large SCL interval accords with the diagram developed in Fig. 1(b), as these alloys are beyond the peritectic reaction and hence not likely to form α-Mg, which precipitates at much lower temperatures. Similar to the Mg–Ag–Ca system, glasses with Mg-content greater than 70 at.% and Yb-content of 7.5 at.% or less begin to show a pronounced exothermic structural relaxation upon approaching the glass transition.

Figure 3(b) shows the corresponding XRD traces, with the halo peak and shape clearly shifting to lower angles of 2θ with increasing Mg-content, particularly between 67.5 and 72.5 at.% Mg. This may correlate with a more specific structural change, which could also be related to the observed structural relaxation prior to *T*_*g*_ and the observed ductility in alloys with >72.5 at% Mg.

Table 3 gives the *Z*_*C*_ and *D*_*C*_ values, and the thermophysical and hardness properties of the Mg–Pd–Ca alloys examined. Here a much more complex liquidus surface is evident compared to those of the Mg–Ag–[Ca,Yb] ternary systems, with the Mg–Pd binary contributing to the creation of two ternary eutectics and multiple peritectic reactions in this region. Due to the smaller radius of Pd, a much closer alignment of packing efficiency between Pd- and Ca-centred clusters is observed, which apart from other chemical or kinetic differences may be a contributing factor to the improved glass-forming ability of this system compared to the Mg–Ag alloys. Here glass-forming maxima are located around Ca-concentrations of 10 at.%, with the largest glass found for Mg_70_Pd_20_Ca_10_, which is an off-eutectic composition but lies directly on the <11> and <17> packing efficiency lines.

**Table 3:** BMG-forming compositions in the Mg-rich region of the Mg–Pd–Ca ternary system, and their associated critical casting sizes, thermophysical properties, and Vickers hardness.

Composition	*Z*_*C*_ (mm)	*D*_*C*_ (mm)	^*^ *T*_*g*_ (°C)	*T*_*X*_ (°C)	*ΔT*_*X*_ (°C)	*T*_*m*_ (°C)	*T*_*l*_ (°C)	*HV*
Mg_75_Pd_15_Ca_10_	0.5	—	117	129	12	499	534	—
Mg_72.5_Pd_15_Ca_12.5_	0.8	1.0	117	134	17	499	544	247
Mg_72.5_Pd_17.5_Ca_10_	1.6	2.0	155	174	19	497	527	217
Mg_72.5_Pd_20_Ca_7.5_	1.4	2.0	163	199	36	504	582	226
Mg_72.5_Pd_22.5_Ca_5_	0.7	1.0	186	203	17	507	620	272
Mg_70_Pd_17.5_Ca_12.5_	1.1	1.5	151	178	27	508	532	216
Mg_70_Pd_20_Ca_10_	2.4	3.0	168	208	40	505	586	225
Mg_70_Pd_22.5_Ca_7.5_	0.9	1.0	183	215	32	502	624	255
Mg_67.5_Pd_22.5_Ca_10_	1.8	2.5	200	223	23	502	625	231
Mg_67.5_Pd_25_Ca_7.5_	0.8	1.0	212	229	17	504	642	267

^*^ *T*_*g*_ values quoted here are the onset temperatures of the glass transition.

**Figure 4:**
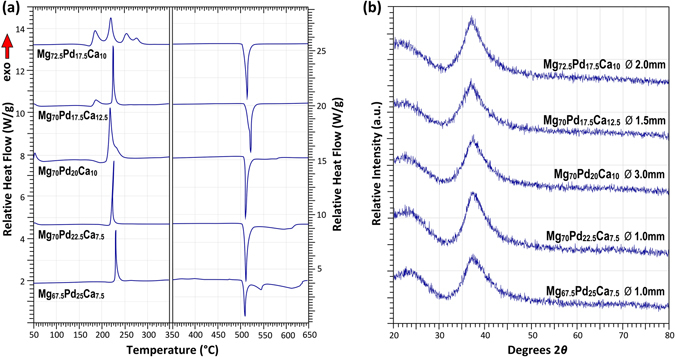
Selected (**a**) DSC and (**b**) XRD traces of Mg–Pd–Ca BMGs.

Figure 4(a) shows selected DSC data of Mg–Pd–Ca BMGs. Strong glass transition signals are evident for alloys with little structural relaxation observed prior to *T*_*g*_. A characteristic of this system is the single crystallisation event witnessed for the alloys with Mg-contents of less than 70 at.% or Ca contents of less than 10 at.%. The crystallisation event for the Mg_70_Pd_22.5_Ca_7.5_ alloy was so rapid and intense that at a heating rate of 20 °C/min the DSC primary crystallisation peak appears curved against the temperature axis, because the reaction heat release has exceeded the time/heating resolution of the DSC. (This result was reproduced several times.). At higher concentrations of Mg, crystallisation events separate and for the Mg_72.5_Pd_17.5_Ca_10_ composition, four individual crystallisation events are evident. The largest glass-former in this system, Mg_70_Pd_20_Ca_10_, also shows the largest SCL interval of 40 °C.

A dramatic drop in *T*_*g*_ and *T*_*X*_ is observed for the Mg_75_Pd_15_Ca_10_ and Mg_72.5_Pd_15_Ca_12.5_ alloys. While Mg_72.5_Pd_15_Ca_12.5_ lies well within the Mg_2_Ca liquidus phase field with reference to Fig. 1(c), Mg_75_Pd_15_Ca_10_ is in close proximity to the ternary eutectic. The dramatic drop in thermal stability may again be due to the incomplete filling of the *β*-site (lack of MRO), because these two alloys have a Pd-content below that topologically required to sustain this (i.e. <16.67 at.%). As also indicated in Fig. 4(a), the melting reactions become more complex as the Mg-content is decreased and Pd content increased. The liquidus data suggest that the majority of these BMGs are located at off-eutectic compositions, some with liquidus intervals in excess of 100 °C. Despite this, GFA remains relatively high. Figure 4(b) shows selected XRD traces for these Mg–Pd–Ca glasses, again exhibiting the characteristic diffuse halo of an amorphous structure.

Table 4 gives the *D*_*C*_ values, and thermophysical and hardness properties of the Mg–Pd–Yb alloys examined. Due to the slightly smaller size of Yb compared to Ca the corresponding efficient packing line lines do not overlap as closely as in Mg–Pd–Ca (see Fig. 1). However, the Mg–Pd–Yb system exhibits a higher maximum critical casting diameter of 4 mm compared to 3 mm in the Mg–Pd–Ca system, with the largest glasses occurring in close proximity to a ternary eutectic reaction and the multiple peritectic reactions shown in Fig. 1(d).

**Table 4:** BMG-forming compositions in the Mg-rich region of the Mg–Pd–Yb ternary system, and their associated critical casting sizes, thermophysical properties, and Vickers hardness.

Composition	*D*_*C*_ (mm)	^*^ *T*_*g*_ (°C)	*T*_*X*_ (°C)	*ΔT*_*X*_ (°C)	*T*_*m*_ (°C)	*T*_*l*_ (°C)	*HV*
Mg_77.5_Pd_15_Yb_7.5_	1.0	151	168	17	475	507	210
Mg_75_Pd_17.5_Yb_7.5_	1.0	155	178	23	472	523	245
Mg_75_Pd_15_Yb_10_	2.0	147	163	16	473	498	242
Mg_73.75_Pd_16.25_Yb_10_	4.0	149	187	38	470	506	264
Mg_72.5_Pd_20_Yb_7.5_	1.0	169	199	30	470	569	279
Mg_72.5_Pd_18.75_Yb_8.75_	2.0	166	194	28	471	536	221
Mg_72.5_Pd_17.5_Yb_10_	3.0	155	191	36	470	513	263
Mg_72.5_Pd_15_Yb_12.5_	1.0	146	165	19	468	508	221
Mg_70_Pd_20_Yb_10_	1.0	172	208	36	466	549	236
Mg_70_Pd_17.5_Yb_12.5_	1.0	160	187	27	471	532	195
Mg_70_Pd_15_Yb_15_	1.0	145	167	22	470	528	238
Mg_67.5_Pd_22.5_Yb_10_	1.0	180	212	32	469	567	198
Mg_67.5_Pd_20_Yb_12.5_	1.0	162	185	23	470	526	220
Mg_67.5_Pd_17.5_Yb_15_	1.0	159	177	18	471	531	192

^*^ *T*_*g*_ values quoted here are the onset temperatures of the glass transition.

**Figure 5:**
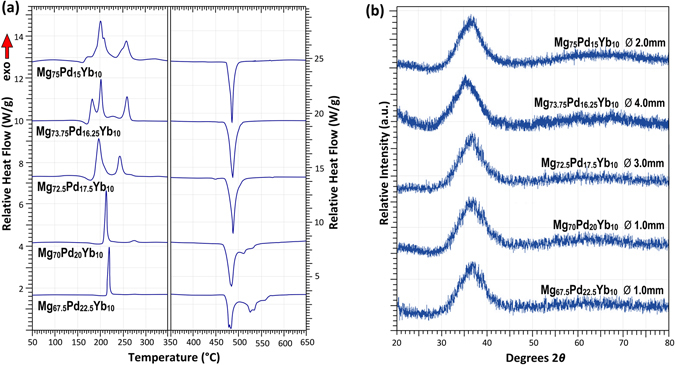
Selected (**a**) DSC and (**b**) XRD traces of Mg–Pd–Yb BMGs.

Figure 5(a) shows selected DSC data of Mg–Pd–Yb BMGs. Similar to the Mg–Pd–Ca system, individual crystallisation reactions become more spread and turn from a single reaction peak to multiple individual peaks with increasing Mg-content. While generally the thermal stability of these alloys is higher than that of the Mg–Ag–Yb system, the glass transition and crystallisation temperatures are slightly lower than those of the Mg–Pd–Ca system. Similar to the Mg–Pd–Ca system, the data in Table 4 illustrate that *T*_*g*_ increases substantially with decreasing Yb-content and increasing Pd-content. Again, those alloys with a Pd-content <16.67 at.% display the lowest *T*_*g*_ values. Despite the high GFA observed in this system, the SCL intervals remain modest compared to those of the Mg–Pd–Ca system. The liquidus data suggest that the larger BMGs are located at near-eutectic or peritectic compositions, with melting reactions becoming more complex for lower Mg concentrations. Figure 5(b) shows corresponding XRD traces for these glasses.

**Figure 6:**
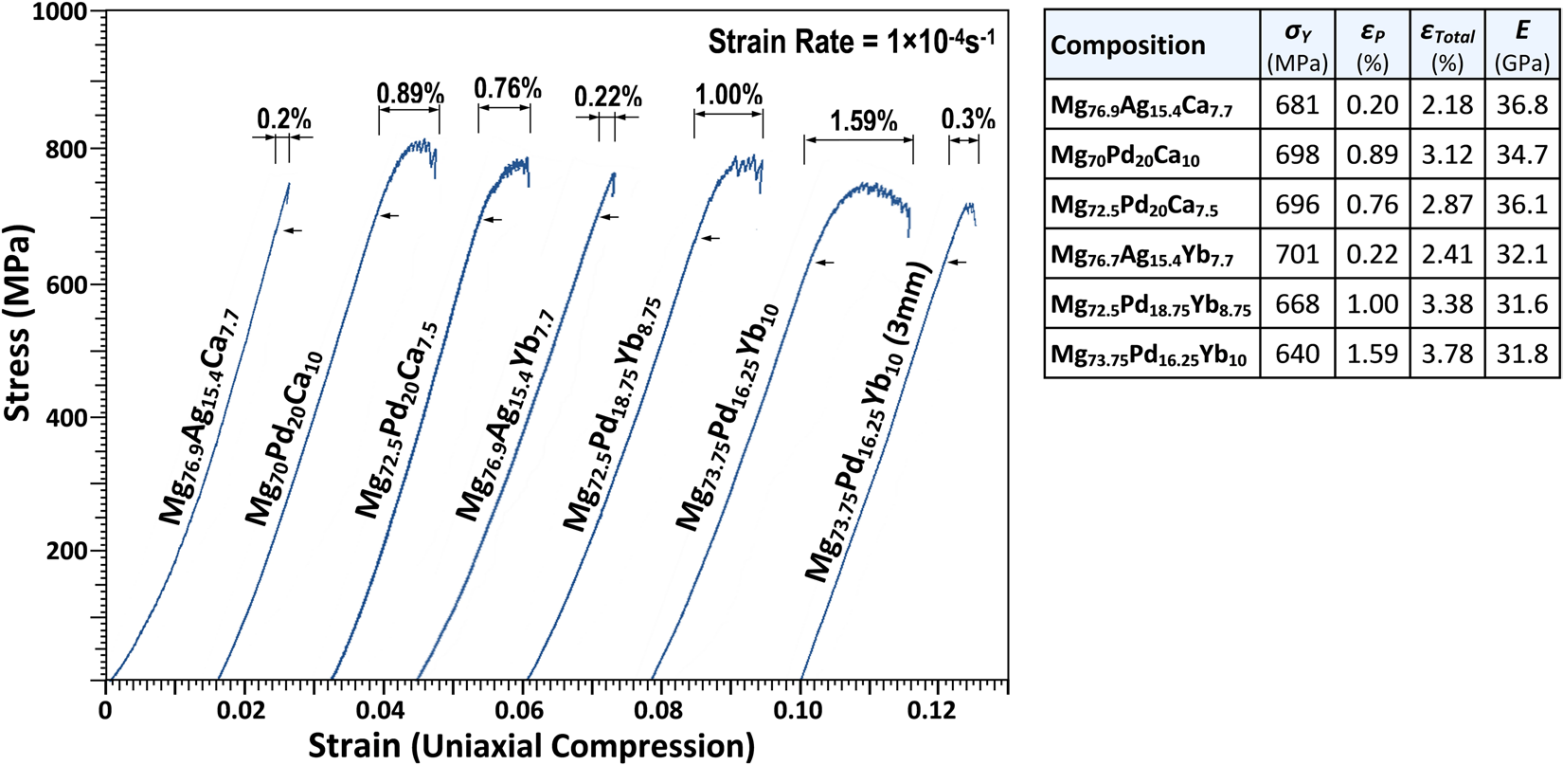
Uniaxial compression curves and associated data for yield strength (σ_y_), plastic strain (ε_P_), total strain to failure (ε_Total_) and Young’s modulus (E) for selected Mg–[Ag,Pd]–[Ca,Yb] BMGs deformed at a strain rate of 1 × 10^−4^ s^−1^. The sample diameters were 1.7 mm (height-to diameter ratio 2:1) with the exception of the Mg_73.75_Pd_16.25_Yb_10_ BMG, which was also tested with a diameter of 3 mm.

Figure 6 shows stress-strain curves of notably ductile alloys in uniaxial compression at room temperature and associated data for yield strength (*σ*_*y*_), plastic strain (*ε*_*P*_), total strain to failure (*ε*_*Total*_) and Young’s modulus (*E*). All BMGs exhibit ultimate yield strengths in excess of 700 MPa. The Mg–Ag–Ca and Mg–Ag–Yb BMGs show limited plasticity of around 0.2% strain. However, the Mg–Pd–Ca and Mg–Pd–Yb BMGs show relatively exceptional room-temperature ductility and stable serrated flow for samples of 1.7 mm in diameter. The Mg–Pd–Yb BMGs appear superior in plastic performance compared to the Mg–Pd–Ca BMGs for the compositions tested here. It is also noted that Mg–Ca-containing BMGs exhibit higher yield strengths and Young’s moduli (engineering stiffness) than the Mg–Yb BMGs, and yield strength was found to be between 2.5 and 3.1 times the Vickers hardness. The Mg_73.75_Pd_16.25_Yb_10_ alloy exhibited the largest plastic strain prior to failure of 1.59%, with a total strain to failure (elastic + plastic) of 3.78% at the sample size of 1.7 mm. Given that this composition had the largest glass-forming ability, samples of 3 mm in diameter were also produced for compression testing; these tests also exhibited serrated flow and the samples deformed plastically up to 0.3% strain, with a total strain to failure of 2.6%. It is well known that sample size dramatically affects shear banding/perceived ductility in metallic glasses^5, 23, 41^, hence it would be expected that these alloys showed further extension of ductility in sample sections of less than 1.7 mm diameter mostly tested here.

## Discussion

### Glass-forming ability

The compositional bulk glass-forming regions found in this work correspond well to the regions predicted by the packing efficiency model, although it should be noted that these regions are quite sensitive to the atomic radius input data. It was found that the critical casting thickness *Z*_*C*_ of the alloys correspond well to their critical casting diameter, *D*_*C*_, following the established geometric cooling rate relationship *Z*_*C*_ = 0.7*D*_*C*_^42^. Glass-forming ability diminished when Ag and Pd-content was <15 at.%, which may be due to the requirements of periodic MRO for Ag- and Pd-centred clusters (i.e. complete filling of *β*-sites). Similarly, GFA dropped at Ca and Yb-content of <7 at.% as compositions then shift from liquidus troughs and ternary eutectics and approach the higher melting point of Mg–Ag and Mg–Pd binary intermetallics. Clearly the Pd-containing glasses outperform the Ag-containing glasses in terms of GFA. Generally speaking, the Mg–Pd BMGs exhibit higher packing efficiency for each atomic species than the Mg–Ag BMGs (note that the packing efficiency lines in Fig. 1 are considerably closer together for Mg–Pd glasses), which may be responsible in part for this observation.

The best glass-former found was the off-eutectic Mg_73.75_Pd_16.25_Yb_10_ alloy with a critical casting diameter of 4 mm, with the Mg_72.5_Pd_17.5_Yb_10_ alloy being the next largest in the Mg–Pd–Yb system at 3 mm. With the ideal Pd concentration for establishing MRO calculated to be 16.67 at.%, a critical casting diameter in excess of 4 mm may exist between these two compositions. Further, an Yb concentration of 11 at.% may also yield greater GFA than alloys with 10 at.%, as this is equivalent to 2 atoms of Yb in a coordination number <17> system (double 5.55 at.%). This is the case with the well-known Mg–Cu–Y, Mg–Cu–Gd and Mg–Cu–Ag–Gd alloys, which see a maximum of GFA when the large atom constituent is 11 at.%^15, 43, 44^.

Interestingly, the best glass-former found in the Mg–Pd–Ca system is Mg_70_Pd_20_Ca_10_ (not at the topologically ideal concentration of 16.67 at.% Pd), at a peritectic rather than eutectic reaction. This follows observations made in other Mg–Ca systems where glass compositions with high GFA are located in close proximity to liquidus lines or peritectic reactions, often far from eutectic reactions^13, 26, 45^. These data further support the idea that liquidus lines, eutectic and peritectic reactions are local compositional indicators of a high structural stability of the liquid often transferred to the vitreous solid upon rapid cooling^26^.

### Thermal stability

Both the Mg–Pd–Yb and Mg–Pd–Ca BMGs exhibit glass transition and crystallisation temperatures considered exceptionally high for Mg-based BMGs (*T*_*g*_ in the range of 146–212 °C and *T*_*x*_ of 163–229 °C), comparable to or exceeding the high-temperature Mg–Ni–RE BMGs^9, 10^. Of the systems studied here, and those reported elsewhere, the Mg–Pd–Ca alloys show the highest glass transition temperatures of all Mg-based BMGs to date, with the Mg_67.5_Pd_25_Ca_7.5_ and Mg_67.5_Pd_22.5_Ca_10_ alloys both having glass transition temperatures in excess of 200 °C. This will likely prove beneficial in enhancing long-term thermal stability and providing resistance to structural relaxation, which can be detrimental to mechanical performance at both room and elevated temperatures over time in Mg-based BMGs^16, 17^. The data in Tables 1–4 suggest that *T*_*g*_ increases substantially with decreasing Ca or Yb-content and increasing Pd- or Ag-content. Here it can be emphasized that the higher melting point of Pd and the enthalpy of bonds that it forms are beneficial in maintaining high glass transition and crystallisation temperatures in these systems. It is noted that the enthalpies associated with crystallisation reactions (DSC data) in the Pd-containing systems are often double of those in Ag-containing systems. This is also a likely contributing factor to the higher GFA observed in the Pd-containing systems. Several BMGs discovered in this work also exhibit large supercooled liquid intervals between the glass transition and crystallisation temperatures, including Mg_70_Pd_20_Ca_10_ (Δ*T*_*X*_ = 40 °C), Mg_70_Ag_22.5_Yb_7.5_ (Δ*T*_*X*_ = 51 °C) and Mg_67.5_Ag_25_Yb_7.5_ (Δ*T*_*X*_ = 57 °C), implying a high resistance to devitrification at elevated temperatures, comparable to that of the well-known Mg–Cu–Y BMGs (Δ*T*_*X*_ = 50–70 °C)^43, 44^. The latter are capable of strains in excess of 1000% when deformed in the SCL region^46, 47^.

### Mechanical performance

It was found that of the alloys tested the Mg–Pd BMGs exhibit superior ductility when compared to the Mg–Ag BMGs, and that the Mg–Pd–Yb BMGs exhibit extended ductility over Mg–Pd–Ca BMGs. In comparison, it is known that the crystalline MgAg intermetallic exhibits ductility based on its electronic bonding state^21^ (and this is also expected, but not reported for Mg–Pd cubic intermetallics). However, it has been postulated that both Ag and Mg, with their excess s electrons, are capable of producing a d-electron band above the Fermi level when bonding with Ca^48^ (again, d-shell bonds have high strength but are highly directional), which in part explains the fact that Ag–Ca and Mg–Ca compounds are brittle as reflected in these findings. This deeper bonding state may also be responsible for the fact that Mg–Ca-containing BMGs exhibit higher yield strength and stiffness than Mg–Yb BMGs.

Generally speaking, the strength/hardness tends to decrease with increasing Mg-content which, based on the bonding arguments presented here, is also expected. It has been shown for other Mg–Ca and Mg–RE glass-forming systems that ductility is highly sensitive to constitution, in that increases in Ca and RE-content substantially reduce the ductility of Mg-based glasses^16^; this has been shown to be an effect of electronic charge transfer^19^. Phenomenologically, the Poisson’s ratio of Pd ~0.39 is considerably higher than that of Ag at ~0.37, and the results in Fig. 6 suggest that the implied chemical bond differences may ultimately affect shear modulus, bulk modulus and shear stability during deformation in these glasses.

In comparison, considerable compressive ductility has been observed in Mg-rich (Mg content >70 at.%) Mg–Ni–Gd BMGs (1 mm diameter), which also have relatively high glass transition and crystallisation temperatures^9^. Substantial ductility has also been reported in Mg-rich (Mg > 85 at.%) Mg–Ni–Ca BMGs (1.2 mm diameter)^14^ and in Mg–Ni–Y amorphous ribbons (20 um thick)^16^, keeping in mind that sample size can affect these results^23, 41^.

### Implications

Based on these findings several Mg-rich BMGs with outstanding combinations of glass-forming ability and thermal and mechanical attributes have been produced. Of these, the Mg_70_Pd_20_Ca_10_ BMG exhibits an exceptional property profile with a high glass-forming ability and critical casting diameter of 3 mm, a high thermal stability with a *T*_*g*_ of 168 °C, a *T*_*X*_ of 208 °C giving a large supercooled liquid/superplastic forming range of Δ*T*_*X*_ = 40 °C, a yield strength of 698 MPa, and considerable room-temperature ductility with an observed plastic strain of 0.89%.

In terms of glass formation, further optimisation of the ternary systems studied here may be achieved. Given the topological/electronic similarities and mutual solid solubility of elements used here, it is anticipated that multi-component combinations stemming from the Mg–[Ag,Pd]–[Ca,Yb] system will likely exhibit extended GFA as entropy is increased.

Based on the emerging trends in reported ductility of Mg-based metallic glasses, extended ductility is possible in Mg-rich glasses (Mg-content >70 at.%) in the presence of group 10 transition metals, i.e. Ni and Pd, which are capable of constituting a full d-shell of electrons in their outermost valence band. Similarly, Mg-based BMGs containing some of the rare-earth metals with d and f electron shells below the Fermi level exhibit superior ductility over Ca additions^9, 16^. Based on these findings and those observed in literature, we anticipate high and tuneable glass ductility and thermal stability in Mg-rich Mg–[Ni,Pd]–[Ca,Yb] alloy systems, or similarly in other Mg–[Ni,Pd]–RE amorphous alloys, which are currently being synthesised by our research group.

## Summary

A range of ductile Mg-rich BMGs from the Mg–Ag–Ca, Mg–Ag–Yb, Mg–Pd–Ca, and Mg–Pd–Yb ternary systems was discovered using a topologically efficient packing model and electron band theory. The Pd-containing alloy systems exhibit glass-forming ability superior to that of the Ag-containing alloys. The Mg–Pd–Ca BMGs exhibit high ductility and the highest thermal stability of any Mg-based BMGs to date, while the Mg–Pd–Yb BMGs exhibit high glass-forming ability and the highest compressive plastic strain to failure reported for a Mg-based BMG. Based on these results, our observations and theoretical analysis, it is expected that ductility will be significantly improved in these and other BMGs if d- and f- band electron interactions can be constrained in participating alloy elements.

## Methods

Nominal alloy compositions (shown in Tables 1–4) were prepared using pure metals: Mg (99.95 wt.%), Ag (99.95 wt.%), Pd (99.95 wt.%), Ca (99.8 wt.%) and Yb (99.99 wt.%). These metals were alloyed in graphite crucibles using an induction furnace in an argon-purged (99.997 wt.%) atmosphere by completely dissolving the solute elements in the molten Mg balance at 750 °C. Samples were produced for each composition by tilt casting the alloys into a wedge-shaped (10:1 taper) copper mould at temperatures of 100 to 150 °C above the liquidus. Compositions of good glass-forming ability were vacuum cast into rods of varying thickness, from 1–4 mm, in 0.5 mm incremental steps where appropriate. The relative glass-forming ability of each alloy was initially determined using electron-based microscopy (Hitachi SU70 SEM) and verified on the central sections of castings using X-ray diffraction (XRD) with a Phillips MRD instrument equipped with a 0.5 mm micro-capillary tube and a Cu Kα radiation source. Thermophysical data were determined by differential scanning calorimetry (DSC) using a Mettler Toledo DSC1 calorimeter at a heating rate of 20 °C min^−1^. For compression testing, samples of 1.7 and 3 mm in diameter with a height-to-diameter ratio of 2:1 were fabricated from as-cast rods. Compression testing was carried out at room temperature using a screw-driven Schenck mechanical testing machine with a compliance of ∼10 nm/N at a strain rate of 10^−4^ s^−1^.

### Data Availability

The datasets generated during and/or analysed during the current study are available from the corresponding author on reasonable request.
